# Transcatheter aortic valve implantation in patients with bicuspid aortic valve stenosis utilizing the next-generation fully retrievable and repositionable valve system: mid-term results from a prospective multicentre registry

**DOI:** 10.1007/s00392-019-01541-8

**Published:** 2019-09-02

**Authors:** Janusz Kochman, Karol Zbroński, Łukasz Kołtowski, Radosław Parma, Andrzej Ochała, Zenon Huczek, Bartosz Rymuza, Radosław Wilimski, Maciej Dąbrowski, Adam Witkowski, Piotr Scisło, Marek Grygier, Maciej Lesiak, Grzegorz Opolski

**Affiliations:** 1 First Department of Cardiology, Medical University of Warsaw, ul. Banacha 1a, PL, 02-097 Warsaw, Poland; 2 Medical University of Silesia, Katowice, Poland; 3 Department of Interventional Cardiology and Angiology, Institute of Cardiology, Warsaw, Poland; 4 Department of Cardiology, Poznan University of Medical Science, Poznan, Poland; 5 Department of Cardiac Surgery, Medical University of Warsaw, Warsaw, Poland

**Keywords:** Transcatheter aortic valve implantation, Bicuspid aortic valve, Lotus™ valve

## Abstract

**Background:**

The aim of this study was to evaluate the outcomes of transcatheter aortic valve implantation (TAVI) in bicuspid aortic valve (BiAV) stenosis using a mechanically expanded Lotus™ device. The prior experience with first-generation devices showed disappointing results mainly due to increased prevalence of aortic regurgitation (AR) that exceeded those observed in tricuspid stenosis.

**Methods and results:**

We collected baseline, in-hospital, 30-day and 2-year follow-up data from a prospective, multicentre registry of patients with BiAV undergoing TAVI using Lotus™ valve. Safety and efficacy endpoints were assessed according to VARC-2 criteria. The study group comprised 24 patients. The mean age was 73.5 years and the mean EuroSCORE 2 was 4.35 ± 2.56%. MDCT analysis revealed Type 1 BiAV in 75% of patients. The mean gradient decreased from 60.1 ± 18.3 to 15 ± 6.4 mm Hg, the AVA increased from 0.6 ± 0.19 to 1.7 ± 0.21 cm^2^. One in-hospital death was observed secondary to aortic perforation. There was no severe AR and the rate of moderate AR equalled 9% at 30 days (*n* = 2). Device success was achieved in 83% and the 30-day safety endpoint was 17%. In the 2-year follow-up, the overall mortality was 12.5% and the 2-year composite clinical efficacy endpoint was met in 25% of the patients (*n* = 6)

**Conclusions:**

The TAVI in selected BiAV patients using the Lotus™ is feasible and characterized by encouraging valve performance and mid-term clinical outcomes.

**Graphic abstract:**

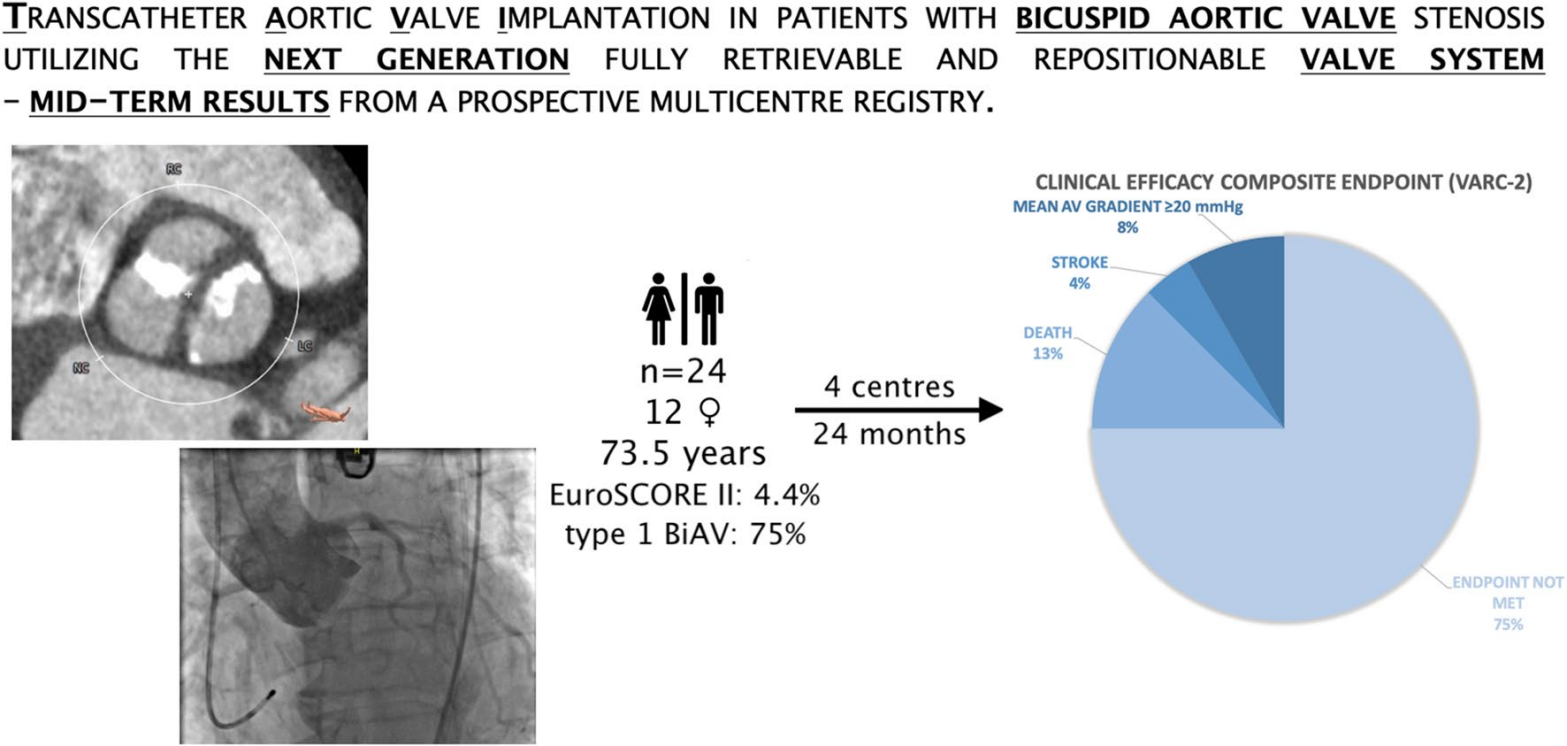

## Introduction

A bicuspid aortic valve (BiAV) is the most common congenital heart defect reported in up to 2% of the general population [1, 2]. The altered anatomy predisposes to a premature calcification and thickening of valve leaflets leading to development of aortic stenosis in large number of patients with BiAV [3]. Although the clinical symptoms occur earlier than in patients with tricuspid valve, BiAV is still an indication for aortic valve replacement (AVR) in significant number of elderly patients. In a recent study, 20% octogenarians who underwent surgical AVR had an underlying BiAV pathology [4]. As these individuals are characterized by an increased operative risk, they might also be suitable candidates for transcatheter aortic valve implantation (TAVI). Although TAVI is a well-established and recommended therapeutic option in high surgical-risk patients, it is still considered as a relative contraindication in BiAV anatomy [5, 6]. The current guidelines on the treatment of valvular disease have omitted this population from their recommendations, as the majority of landmark clinical trials excluded those with BiAV stenosis [7]. Unfavourable anatomy with heavily calcified and asymmetrical aortic valve cusps accompanied by annular eccentricity and aortopathy were perceived as features that could lead to prosthesis dysfunction and periprocedural complications. These concerns were further strengthened by results of few small TAVI registries that showed a higher rate of moderate-to-severe aortic regurgitation (AR) than in tricuspid valves [8–11]. The latter is probably associated with important drawbacks of the first-generation devices such as lack of dedicated sealing systems and limited ability to reposition the bioprosthesis. The new-generation Lotus™ Valve System (Boston Scientific, MA, USA) was designed to overcome the aforementioned restrictions. It adopts a unique mechanical expansion mechanism and is made of a single-braided nitinol wire and three bovine pericardial leaflets. The device is fully repositionable and resheatable even in the expanded position, before final release. It is equipped with a surrounding flexible membrane designed to seal the paravalvular gaps between the prosthesis and native annulus, which aims to reduce the risk of significant paravalvular leaks (PVLs) [12–14]. This was confirmed in the REPRISE II trial in which the rate of moderate PVL was below 2% [15]. However, the data regarding Lotus performance in BiAV patients are scarce. To address this important clinical issue, we have performed safety and efficacy analysis of TAVI in patients with BiAV stenosis treated with Lotus™ valve implantation.

## Methodology

The study was based on a prospective registry of patients with BiAV undergoing TAVI using Lotus™ valve in four academic centres in Poland. Patients entered into the registry were to meet the generally accepted criteria for TAVI, which included an intermediate or high surgical risk, end-stage renal failure, chronic pulmonary disease, pulmonary hypertension or other contraindications to surgical aortic valve replacement (SAVR) not included in the risk scores (such as “porcelain” aorta, previous chest radiotherapy, previous pulmonary lobectomy, cirrhosis with portal hypertension, previous chest surgery, cognitive dysfunction due to neurological disease). Severe aortic stenosis was defined as an aortic valve area (AVA) ≤ 1 cm^2^ or indexed AVA ≤ 0.6 cm^2^/m^2^, or mean aortic gradient ≥ 40 mm Hg or an aortic velocity ≥ 4 m/s in the presence of clinical symptoms. The database contains detailed demographic and clinical characteristics, results of imaging studies, including echocardiography and multi-detector computed tomography (MDCT), laboratory assessment, procedural outcomes and the results of a short-term and mid-term follow-up. The treating institutions followed patients prospectively using clinical and echocardiographic evaluations.

All patients included in the analysis underwent contrast-enhanced, ECG-gated MDCT imaging using contemporary CT systems according to the local institutional CT scan protocols. The operators used the images for valve sizing and procedure planning. For the purpose of this analysis, the MDCT source data were collected and retrospectively re-evaluated by one experienced CT analyst using the 3Mensio valve analysis software (3Mensio Medical Imaging, Bithoven, The Netherlands). The annular plane was identified as the short axis through the nadir of each coronary cusps; and the diameter, perimeter and area were measured. Additional measurements were taken at the intercommissural distance − 4 mm above the annular plane, at the level of the ventricular outflow tract (4 mm below the annular plane), sinus of Valsalva, and ascending aorta. The heights of both coronary ostia were also recorded. The eccentricity index of annular plane was calculated using the formula 1 – (minimal diameter/maximal diameter). Additionally, the degree of oversizing was derived from the annular plane perimeter (perimeter oversizing = (device perimeter – annular perimeter)/annular perimeter × 100) and annular plane area (area oversizing = (device area – annular area)/annular area × 100). BiAV morphology was described using the Sievers classification, which takes into account the number of cusps, the presence and spatial distribution of raphes [16]. The Type 0 was identified in the case of two fully developed cusps with one commissure and no raphe; Type 1 was characterized by one completely developed cusp and two smaller malformed cusps fused by one raphe; and Type 2 was assigned when two raphes were present. The functional BiAV was classified as a tricuspid valve with evident symmetry of all three cusps, secondarily fused by a degenerative process and no evidence of raphe. Further subcategories were reported according to the location of the raphe: R–L, R–N, N–L (R: right, L: left, N: non-coronary). Based on the previous literature, any raphe below 3 mm long was considered as non-significant [4].

Pre-procedural, discharge, 30-day and 2-year echocardiographic results were entered by the participating centres and included the aortic annulus, aortic root and ascending aorta diameters, the aortic valve area, the peak/mean transvalvular gradient, the extent and distribution of valve calcifications, mitral and aortic valve regurgitation, mean transprosthetic gradient, and effective orifice area. AR after TAVI was defined as the sum of transvalvular and paravalvular regurgitation. Additionally, the paravalvular regurgitation was analysed separately. The AR was classified as none/trivial, mild, moderate or severe.

The technical aspects of TAVI have been described previously [17, 18]. Briefly, all procedures were performed under general anesthesia or conscious sedation either in dedicated hybrid rooms or in cardiac catheterization laboratories. All patients underwent TAVI via the transfemoral approach; the vascular access was secured using either a Prostar 10 French XL (Abbott Vascular Device, Redwood City, California) or surgical technique, depending on the local protocols. A 20-French Lotus™ Introducer (Boston Scientific, MA, USA) was used for 23-mm Lotus™ valves and 22-French for those who received 25-mm and 27-mm valves. Balloon valvuloplasty with an undersized balloon was performed at the discretion of the operator. The positioning and deployment of Lotus™ valves strictly followed the manufacture’s recommendations and were carried out based upon the best clinical practice [13, 15]. A control angiography was done before final release of the valve to assess the appropriate positioning, the degree of AR and patency of coronary arteries. In case of suboptimal result, the valve was repositioned, and the aortography was repeated to ensure that no further manipulation was needed and to confirm a good final result.

The standard post-procedural care included observation at the intensive care unit for at least 24 h and duration was mainly related to post-procedural complications. A dual antiplatelet therapy was initiated in all patients comprising a life-long 75 mg of acetylsalicylic acid and 3 months of 75 mg of clopidogrel. In case of indications for oral anticoagulant, the decision regarding the choice of the molecule, dosage and duration was left to the decision of local heart team. Before discharge, all patients underwent transthoracic echocardiography. The patients were scheduled for a 30-day and a 24-month follow-up visits to collect clinical and echocardiographical data. The data were prospectively collected and entered into the registry.

The main endpoints of the study were device success and 30-day safety composite endpoint as defined by the VARC-2 criteria [19]. The secondary endpoint was 2-year all-cause mortality and 2-year composite clinical efficacy endpoint [as defined by the VARC-2 criteria). Device success comprised of the absence of procedural mortality, successful implantation of a single prosthesis with its appropriate placement and function (no severe prosthesis-patient mismatch (< 0.65 cm^2^/m^2^), mean aortic valve gradient < 20 mmHg or peak velocity < 3 m/s, no moderate/severe PVL] and successful retrieval of the delivery system*.* The 30-day safety composite endpoint included all-cause mortality, stroke/TIA, life-threatening bleeding, acute kidney injury (stage 2 or 3), coronary artery obstruction requiring intervention, major vascular complication and valve-related dysfunction requiring repeat procedure (BiAV, TAVI or SAVR). The 2-year clinical efficacy endpoint consisted of all-cause mortality, stroke, requiring hospitalizations for valve-related symptoms or worsening congestive heart failure, NYHA class III or IV and valve-related dysfunction.

Quantitative data are presented as mean and standard deviation (SD) or as median (interquartile range) and qualitative variables as numbers and percentages. An unpaired Student’s *t* test or Wilcoxon rank-sum test was used for comparison of quantitative variables, whereas the comparison of qualitative variables was performed with the two-tailed Fisher’s exact test. Statistical significance was defined as *p* < 0.05. Statistical analysis was performed using Medcalc ver.11 and Statistica ver. 12.

## Results

Between March 2015 and December 2016, 24 patients met the inclusion criteria of the prospective registry and were included in the study. Baseline demographics and clinical characteristics are presented in Table 1. Briefly, the mean age was 75.3 years with equally distributed gender. Patients had moderate surgical risk and the majority of them demonstrated heart failure with NYHA III/IV symptoms. Detailed assessment of aortic stenosis severity and type of BiAV morphology are depicted in Table 2. The average mean gradient was 60.1 ± 18.31 mm Hg, the mean AVA was 0.6 ± 0.19 cm^2^. As far as the valve morphology is considered, Type 1 was the most frequently identified. Moderate/severe degree of valve calcification was recognized in majority of patients.

**Table 1:** Baseline characteristics (*n* = 24)

Age, years	75.3 ± 7.85
Female	12 (50)
Logistic Euroscore (%)	13.4 ± 10.39
Euroscore 2 (%)	4.35 ± 2.56
Height, cm	162.5 ± 6.83
BMI, kg/m^2^	28.0 ± 4.65
NYHA functional class	
NYHA I	3 (13)
NYHA II	6 (25)
NYHA III or IV	15 (63)
Diabetes mellitus type 2	5 (21)
Atrial fibrillation	6 (25)
Hypertension	16 (67)
Coronary artery disease	
None	11 (45)
CCS I or II	13 (55)
Previous myocardial infarction	4 (17)
Previous PCI	1 (4)
Previous CABG	2 (8)
Stroke/intracranial bleeding	3 (13)
Previous pacemaker	4 (17)
Peripheral artery disease	4 (17)
COPD	7 (29)
Pulmonary hypertension	2 (8)
eGFR, mL/min	64.4 ± 16.51

Values are mean ± SD or *n* (%)

*CCS* Canadian cardiology scale, *CABG* coronary artery bypass grafting, *COPD* chronic obstructive pulmonary disease, *eGFR* estimated glomerular filtration rate, *MI* myocardial infarction, *NYHA* New York Heart Association

**Table 2:** Pre-procedural echocardiographic and computed tomographic imaging assessment (*n* = 24)

Transthoracic echocardiography	
AVA, cm^2^	0.6 ± 0.19
AVA indexed, cm^2^/m^2^	0.3 ± 0.12
Maximum velocity, m/s	4.9 ± 0.87
Mean gradient, mm Hg	60.1 ± 18.31
Ejection fraction, % ≤ 30%	50 ± 2,9 4 (17)
Pulmonary artery pressure, mm Hg	42 ± 16.5
Mitral regurgitation	
None/trivial	2 (8)
Mild	15 (63)
Moderate	7 (29)
Severe	0 (0)
Tricuspid regurgitation	
None/trivial	3 (13)
Mild	13 (54)
Moderate	7 (29)
Severe	1 (4)
Aortic regurgitation	
None/trivial	9 (38)
Mild	11 (46)
Moderate	3 (13)
Severe	1 (4)
Multi-detector computed tomography	
Annular plane	
Minimal diameter, mm	22 ± 2.9
Maximal diameter, mm	28 ± 3.0
Eccentricity index	0.21 ± 0,92
Perimeter, mm	77 ± 4.8
Area, cm^2^	0.5 ± 0.06
Area-derived diameter, mm	25 ± 2.6
Perimeter-derived diameter, mm	26 ± 2.6
Intercommissural distance 4 mm above the plane, mm	25 ± 3.9
Left ventricular outflow tract	
Minimal diameter, mm	22 ± 3,5
Maximal diameter, mm	30 ± 3.4
Eccentricity index	0.28 ± 0.125
Perimeter, mm	84 ± 9.7
Area, cm^2^	0,5 ± 1.24
Area-derived diameter, mm	25 ± 3.0
Perimeter-derived diameter, mm	27 ± 3,1
Calcium scoring, mm^3^	1779 ± 819.1
Distance of annulus to ostia of coronary arteries, mm	
Left ostium	14 ± 3.2
Right ostium	16 ± 2.5
Diameter of the ascending aorta	38 ± 4.6
Bicuspid valve types	
Type 0	2 (8)
Type 1	18 (75)
Left–right	15 (63)
Right–noncoronary	2 (8)
Left–noncoronary	1 (4)
Type 2	0 (0)
Functional	2 (8)
Undetermined	2 (8)

Values are mean ± SD, mean ± SD (minimum, maximum) or n (%)

Eccentricity index was determined using the formula: 1 – (minimal diameter/maximal diameter)

Perimeter oversizing was determined using the formula [(device perimeter – annular perimeter)/annular perimeter × 100]

Area oversizing was determined using the formula [(device area – annular area)/annular area × 100]

TAVI was performed via femoral route in all patients, in 46%, the procedure was done with local anaesthesia and conscious sedation. Pre-implantation balloon valvuloplasty was conducted in majority of patients (*n* = 15, 63%). The most frequently used valve was the Lotus™ 25 mm. The repositioning/resheathing feature was used in 42% (ten patients) of the procedures, of which in two cases (8.3%), complete prosthesis retrieval was required. The immediate angiographic assessment showed no severe AR (AR), in one case, a moderate AR was identified. The detailed procedural characteristics are depicted in Table 3.

**Table 3:** Procedural characteristics (*n* = 23)

Lotus valve size	
23 mm	8 (35)
25 mm	10 (43)
27 mm	5 (22)
Balloon predilatation	15 (65)
Aortic regurgitation by angiography	
None/trivial	15 (65)
Mild	7 (30)
Moderate	1 (4)
Severe	0 (0)
Device oversizing, %	
Perimeter	− 2.9 ± 7.13
Area	− 1.3 ± 14.46
Contrast media, mL	132 ± 64.2
Duration of procedure, min	139 ± 79.0
Fluoroscopy duration, min	35 ± 15.1
Radiation dose, mGy	1587 ± 1098.7

Percentage calculated for 23 patients, as in 1 patient, the valve was not implanted due to abdominal aorta perforation and need for urgent abdominal surgery

**Fig. 1:**
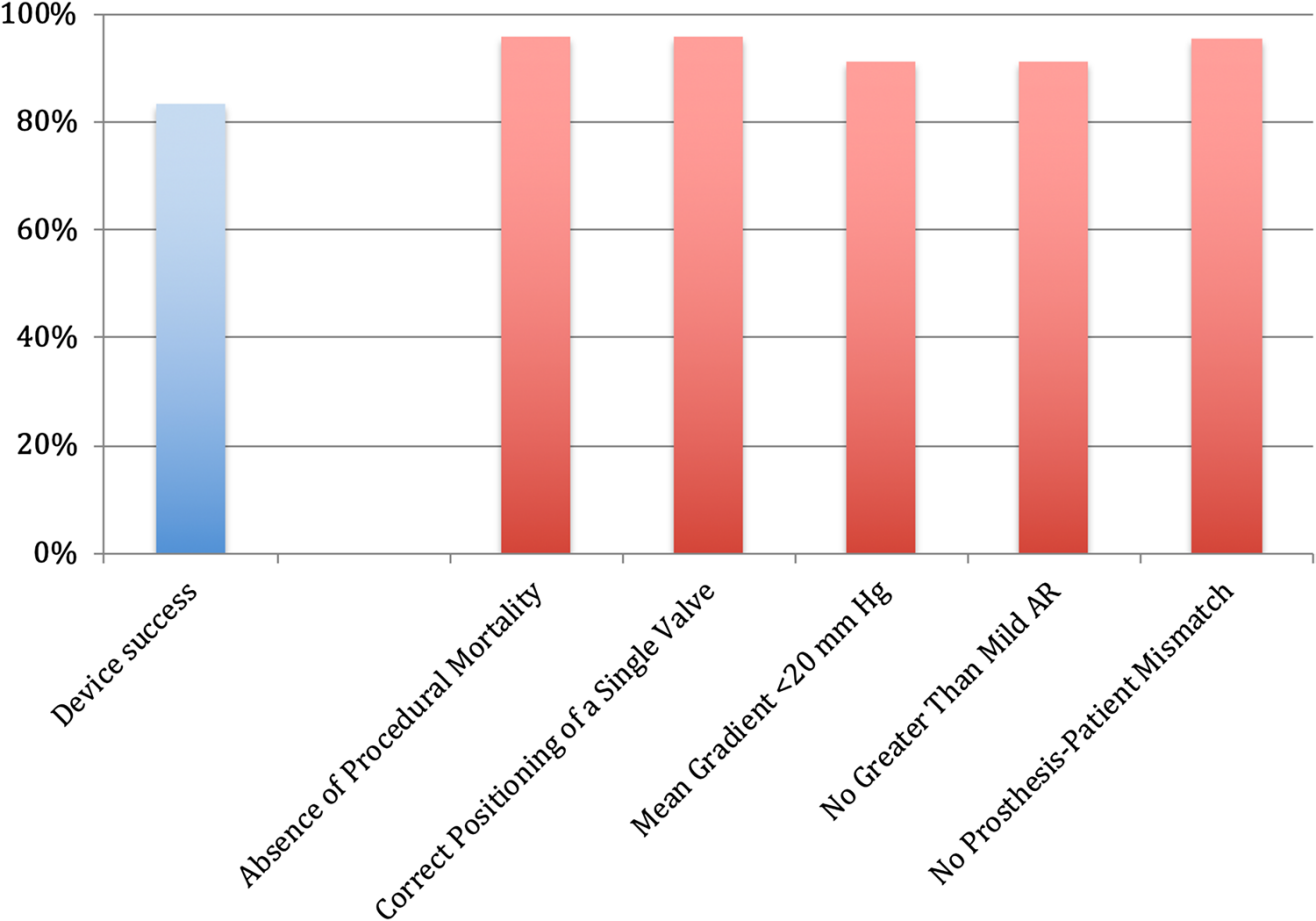
Device success and composites of Lotus valve implantation. Primary outcome measure of Valve Academic Research Consortium 2-defined device success and its composites. *AR* aortic regurgitation

The VARC-2-defined device success was achieved in 83% of patients (*n* = 20); the detailed breakdown of its composites is presented in Fig. 1.

There were no conversions to open-heart surgery. One patient required urgent abdominal surgery due to perforation of the tortuous abdominal aorta that was noted soon after insertion of the Lotus introducer sheath, which was complicated by cardiogenic shock and multi-organ failure resulting in death on day 7.

Any type of VARC-2 bleeding and vascular complications occurred in 29.2% (*n* = 7) and 25% (*n* = 6), respectively. Life-threatening and major bleedings occurred in three patients (13%) and all were directly related to the procedure. There were two overt access site bleedings, and one caused by the above-mentioned abdominal aorta perforation. Major vascular complications were found in three subjects. Of which, two haematomas and one aforementioned aortic perforation were diagnosed and required blood transfusion of at least four units.

There was one minor stroke diagnosed 1 day after the procedure with full recovery within next 72 h (due to limited availability and lack of reimbursement, cerebral protection was not implemented in any of the procedures). The pre-discharge echocardiographic assessment showed significant reduction of the mean gradient from 60.1 ± 18.3 to 15 ± 6.4 mm Hg and increase in the AVA from 0.6 ± 0.19 to 1.7 ± 0.21 cm^2^. There was no severe AR; in two patients (9%), a moderate regurgitation was found (Table 4).

**Table 4:** Procedural and in-hospital clinical outcomes

Procedural and clinical outcomes (*n* = 24)	
Conversion to cardiothoracic surgery	0 (0)
Valve migration	0 (0)
Coronary obstruction	0 (0)
Cardiac tamponade	0 (0)
Bleeding	
Life-threatening/disabling	1 (4)
Major	2 (8)
Minor	
Blood transfer (≥ 4 units)	3 (13)
MI	1 (4)
Stroke/TIA/RIND	1 (4)
Vascular complications	
Major	3 (13)
Minor	3 (13)
Acute kidney injury	1 (4)
Permanent pacemaker implantation	6 (30^a^)
Successful valve implantation	23 (96)
Hospital stay, days	10 ± 5.3
Echocardiographic outcomes (*n* = 23^b^)	
Ejection fraction, %	51 ± 11.2
AVA, cm^2^	1.7 ± 0.21
AVA indexed, cm^2^/m^2^	0.85 ± 0.175
Mean gradient, mm Hg	15 ± 6.4
Maximal velocity, m/s	2.7 ± 0.64
Aortic regurgitation	
None/trivial	16 (70)
Mild	5 (22)
Moderate	2 (9)
Severe	0 (0)
Paraprosthetic aortic regurgitation None/trivial Mild Moderate Severe	17 (74) 4 (17) 2 (9) 0 (0)
Mitral regurgitation None/trivial Mild Moderate Severe	6 (26) 14 (61) 3 (13) 0 (0)
Pulmonary artery pressure, mm Hg	43 ± 15.9

^a^Percentage calculated for 20 patients, as 4 patient had previous pacemaker

^b^Percentage calculated for 23 patients, as 1 patient did not undergo valve implantation due to abdominal aorta perforation and need for urgent abdominal surgery

The 30-day safety composite end point was achieved in 17% (*n* = 4); a detailed breakdown of the composites is presented in Table 5. There were neither any post-discharge deaths, episodes of new bleeding nor vascular complications. Seven patients (35%) required new pacemaker implantation for advanced conduction disturbances.

**Table 5:** 30-day clinical and echocardiographic outcomes

Clinical outcomes (*n* = 24)	
All-cause mortality	1 (4)
Stroke disabling and non-disabling	1 (4)
MI	0 (0)
Bleeding complications	
Life-threatening/disabling	1 (4)
Major	2 (8)
Minor	4 (17)
Vascular complications	
Major	3 (13)
Minor	3 (13)
Acute kidney injury	1 (4)
Permanent pacemaker implantation	7 (35^a^)
Echocardiographic outcomes (*n* = 23^b^)	
Ejection fraction, %	53 ± 12.5
Aortic valve area, cm^2^	1.7 ± 0.16
Mean gradient, mm Hg	14 ± 4.3
Maximum velocity, m/s	2.3 ± 0.44
Aortic regurgitation	
None/trivial	16 (70)
Mild	5 (22)
Moderate	2 (9)
Severe	0 (0)
Paraprosthetic aortic regurgitation	
None/trivial	17 (74)
Mild	4 (17)
Moderate	2 (9)
Severe	0 (0)
Mitral regurgitation	
None/trivial	6 (26)
Mild	14 (61)
Moderate	3 (13)
Severe	0 (0)
Pulmonary artery pressure, mm Hg	45 ± 16.9

^a^Percentage calculated for 20 patients, as 4 patient had previous pacemaker

^b^Values calculated for 23 patients as 1 patient died

In the 2-year follow-up, additional two deaths were reported resulting in overall mortality of 12.5%. The 2-year composite clinical efficacy endpoint was met in 25% of the patients (*n* = 6) (Table 6).

**Table 6:** Two-year clinical and echocardiographic outcomes

Clinical outcomes (*n* = 24)	
All-cause mortality	3 (12.5)
Clinical efficacy according to VARC-2	6 (25)
Stroke disabling and non-disabling	1 (4.2)
MI	0 (0)
Bleeding complications	
Life-threatening/disabling	1 (4.2)
Major	2 (8.3)
Minor	4 (16.6)
Vascular complications	
Major	3 (12.5)
Minor	3 (12.5)
Acute kidney injury	1 (4.2)
Permanent pacemaker implantation	7 (35^a^)
Echocardiographic outcomes (*n* = 21^b^)	
AVA, cm^2^	2.0 ± 0.5
AVA indexed, cm^2^/m^2^	1.2 ± 0.3
Maximum velocity, m/s	2.6 ± 0.5
Mean gradient, mm Hg	15.8 ± 10
Ejection fraction, %	51.9 ± 11
≤ 30%	0 (0)
Mitral regurgitation	
None/trivial	16 (76.2)
Mild	4 (19)
Moderate	1 (4.8)
Severe	0 (0)
Tricuspid regurgitation	
None/trivial	18 (85.7)
Mild	2 (9.5)
Moderate	1 (4.8)
Severe	0 (0)
Aortic regurgitation	
None/trivial	20 (95)
Mild	1 (4.8)
Moderate	0 (0)
Severe	0 (0)

^a^Percentage calculated for 20 patients, as 4 patient had previous pacemaker

^b^Values calculated for 21 patients as 3 patients have died

## Discussion

To the best of our knowledge, this is the first study presenting 2-year outcomes of TAVI in a consecutive multicentre series of patients with bicuspid aortic valve stenosis treated with new-generation Lotus™ valve. The system was first certified and introduced in March 2015; however, due to technical difficulties with the locking mechanism, it has been temporarily withdrawn from the market and the second reiteration (Lotus Edge™) was introduced in December 2018. However, despite using the previous version, we have demonstrated that implantation of this bioprosthesis in a challenging BiAV anatomy is safe and feasible resulting in favorable valve performance and low incidence of significant PVLs. The latter finding is of particular interest as prior experience with first-generation valves revealed high risk of AR in patients with BiAV stenosis [10, 20, 21]. In the recent multicentre, patient-level meta-analysis comprising 108 patients, the rate of moderate and severe AR was 30.8% at 30 days [20]. Similar frequency of significant AR (32%) in BiAV patients treated with first-generation devices was previously reported by our group [10]. These outcomes may raise some concerns, as there is clear evidence that significant AR is associated with an increased late mortality [6, 22]. In the present study, there was no severe AR, whereas moderate was found in only two patients (8%). There is number of factors that could potentially explain these favorable outcomes, among which the inherent properties of the Lotus™ device seem to play an important role. The ability to resheath and reposition the bioprosthesis at any stage of the deployment process, which was utilized in nearly half of our patients, facilitates accurate and precise positioning, and allows for final assessment of the fully functional state before disconnecting the delivery system. Moreover, implantation does not require rapid pacing assuring haemodynamic stability throughout the procedure. Additionally, the adaptive seal may reduce degree of PVLs by filling the gaps between the prosthesis and the native valve. This recently published REPRISE II study confirmed the low risk of post-implantation AR in tricuspid aortic valve anatomy [15]. Although the results are promising, it remains unknown whether the decreased rates of moderate and severe ARs observed with the Lotus™ device will have an impact on the long-term mortality of patients with BiAV stenosis.

The sizing of the bioprosthesis based on MDCT that was utilized in all our patients is another important factor proved to have a positive impact on the rate of device success. As shown in the largest published registry on TAVI in BiAV, the utilization of MDCT resulted in more than 50% reduction of significant post-procedural ARs [8]. Additionally, the diagnostic value of this imaging technique in comparison with echocardiography has a higher sensitivity and specificity for identification of BiAV, especially in Type 1, in which raphe could be overshadowed by calcifications [23].

The detailed analysis of procedural data revealed some degree of device undersizing, which causes are not fully understood. Traditionally profound undersizing was considered to be associated with higher incidence of AR and device embolization, therefore, was contraindicated. This recommendation was based on experience from tricuspid anatomy patients and thus not necessarily applicable to patients with different anatomy. In fact, in our cohort, the degree of AR was low despite an evident undersizing. One may speculate that a more aggressive approach and implantation of larger valves would have resulted in even lower risk of post-procedural AR, as it has been shown that oversizing can minimize the risk of significant AR [24]. On the other hand, excessive oversizing may cause aortic root rupture/haematoma [25], coronary obstruction [26], or atrioventricular block [27, 28]. Additionally, the specific BiAV anatomy characterized by high leaflet coaptiation, extensive asymmetric calcifications and ellipticity of the annulus can lead to incomplete and asymmetric valve expansion, which may impact the acute valve performance and its long-term durability. Therefore, it has been suggested that valve sizing in bileaflet anatomy might require different sizing approach than that currently used in tricuspid valves. Namely, it should not be based solely on the annular but rather on supra-annular measurements taking into account the intracommissural distance. The observed device undersizing in our registry suggests that operators applied this new strategy [29].

It should also be taken into account that the new-generation valves are equipped with sealing cuff that could further overcome the previously observed drawbacks of undersizing. The recently published data regarding the new-generation balloon-expandable Sapien 3 valve in patients with large annuli exceeding the indicated dimensions, confirmed excellent results with no moderate or severe AR despite the inevitable undersizing [30]. These results were further supported by another analysis of Sapien 3, which showed that a low degree of MDCT area oversizing (< 5%) is associated with decreased rates of AR [28]. To validate this hypothesis-generating findings, a dedicated study with new-generation devices and a revised annulus-sizing algorithm would be needed.

The presented device success in our cohort was relatively high (83%), despite the adverse anatomic characteristics typical for bicuspid patients and was comparable to that reported in tricuspid valves. It should be noticed that in our study, the VARC-2 criteria were utilized, whereas most of the published outcomes are based on the first definition of VARC, which does not take into account the prosthesis-patient mismatch. The recent publication with Lotus™ valve in tricuspid aortic stenosis showed 84% device success based on VARC-2 criteria [31]. We did not observe any valve embolization, coronary obstruction or need for surgical aortic valve replacement, and the 30-day composite safety end point was similar to that observed in tricuspid valve populations [32]. Of note, despite the use of larger sheath sizes, the rates of bleedings as well as vascular complications were not significantly higher as compared to outcomes from other new-generation devices. This can partially be explained by appropriate patient selection, especially with respect to the femoral artery size. It should be noted that the 30-day mortality was 4%, which seems relatively high taking into account the moderate risk profile of our cohort, but results from a relatively substantial impact of the one death on the overall mortality. Furthermore, in the 2-year follow-up, the all-cause mortality was 12.5%, which is well within the published literature [21]. New pacemaker implantation was performed in seven patients (35%), which is in the range of the prior results of TAVI in BiAV utilizing first-generation devices (14–50%) and is similar to the rate observed in tricuspid valves treated with the Lotus™ prosthesis (29%) [8–11, 10, 11]. The freedom from the clinical efficacy composite endpoint after 2 years was observed in 75% of the study population—a result which is difficult to compare with the previously published reports, due to the scarcity of a long-term follow-up based on VARC-2 criteria in this cohort of patients in the currently available literature. In the already-mentioned study by Yousef et al. after 1 year of follow-up, the all-cause mortality was 16.9%, 4.3% of patients remained heavily symptomatic (NYHA class III–IV) and 27.7% had AR ≥   2 + [20]. In one of the largest analyses of new-generation valves in BiAV [21], which included 11 patients treated with Lotus™ device, 30-day mortality in that subpopulation was 9.1%, while the early safety endpoint was met in 18.2% (due to one death and one major vascular complication). No significant differences in VARC-2-defined endpoints at 30 days between the Lotus™ valve and the remaining prostheses were observed. Overall, new-generation devices were associated with less paravalvular regurgitation. Of note, all-cause mortality of the entire group was 14.4% at 1 year, which is in line with our findings. In another recently published study—RESPOND—31 patients with BiAV had undergone Lotus™ valve implantation. One death and one stroke were observed in 30-day follow-up. There were no cases of moderate or severe PVL and the pacemaker rate was 22.2% [33]. Again, the mid-term mortality (9.7% at 1 year) was in agreement with our results.

### Study limitations

The main limitation of our study is the lack of randomization with all inherent restrictions of this type of study design. Second, we couldn't exclude a bias related to relative weight of each centre that may limit the generalizability of outcomes. Third, the echocardiographic findings, especially the post-procedural AR, were not assessed by an independent core laboratory, which might have impacted the reported outcomes, as these parameters are operator dependent. Fourth, the differentiation between functional and congenital bicuspid valves is difficult, therefore, we cannot exclude some degree of misclassification in this respect. Fifth, as the registry includes only patients who underwent TAVI procedure, this could have potentially lead to a selection bias and thus the presented results should be interpreted with caution.

## Conclusions

In conclusion, the TAVI in selected BiAV patients using the Lotus™ is feasible and characterized by encouraging valve performance and clinical outcomes. It should be noted that the presented cohort was of a moderate surgical risk as described by the low EuroSCORE 2, therefore, these findings should be viewed with caution when discussing high-risk patients. Although the overall number of analysed subjects is low, the study sample should not be regarded as negligible especially in view of the inadequate body of evidence currently available in the literature.

### Impact on daily practice

We have shown that implantation of the second generation, repositionable and retrievable, transcatheter valve system in bicuspid aortic valve anatomy is safe and efficient in selected patients; these results may impact the current contraindications for TAVI.

## References

[1] Siu SC, Silversides CK (2010). Bicuspid aortic valve disease. J Am Coll Cardiol.

[2] Nistri S, Basso C, Marzari C, Mormino P, Thiene G (2005). Frequency of bicuspid aortic valve in young male conscripts by echocardiogram. Am J Cardiol.

[3] Reardon MJ, Adams DH, Kleiman NS, Yakubov SJ, Coselli JS, Deeb GM, Gleason TG, Lee JS, Hermiller JB, Chetcuti S, Heiser J, Merhi W, Zorn GL, Tadros P, Robinson N, Petrossian G, Hughes GC, Harrison JK, Maini B, Mumtaz M, Conte JV, Resar JR, Aharonian V, Pfeffer T, Oh JK, Qiao H, Popma JJ (2015). 2-year outcomes in patients undergoing surgical or self-expanding transcatheter aortic valve replacement. J Am Coll Cardiol.

[4] Roberts WC, Ko JM (2005). Frequency by decades of unicuspid, bicuspid, and tricuspid aortic valves in adults having isolated aortic valve replacement for aortic stenosis, with or without associated aortic regurgitation. Circulation.

[5] Leon MB, Smith CR, Mack M, Miller DC, Moses JW, Svensson LG, Tuzcu EM, Webb JG, Fontana GP, Makkar RR, Brown DL, Block PC, Guyton RA, Pichard AD, Bavaria JE, Herrmann HC, Douglas PS, Petersen JL, Akin JJ, Anderson WN, Wang D, Pocock S, Trial Investigators PARTNER (2010). Transcatheter aortic-valve implantation for aortic stenosis in patients who cannot undergo surgery. N Engl J Med.

[6] Smith CR, Leon MB, Mack MJ, Miller DC, Moses JW, Svensson LG, Tuzcu EM, Webb JG, Fontana GP, Makkar RR, Williams M, Dewey T, Kapadia S, Babaliaros V, Thourani VH, Corso P, Pichard AD, Bavaria JE, Herrmann HC, Akin JJ, Anderson WN, Wang D, Pocock SJ, Trial Investigators PARTNER (2011). Transcatheter versus surgical aortic-valve replacement in high-risk patients. N Engl J Med.

[7] Nishimura RA, Otto CM, Bonow RO, Carabello BA, Erwin JP, Guyton RA, O'Gara PT, Ruiz CE, Skubas NJ, Sorajja P, Sundt TM, Thomas JD, ACC, AHA Task Force Members (2014). 2014 AHA/ACC Guideline for the Management of Patients With Valvular Heart Disease: executive summary: a report of the American College of Cardiology/American Heart Association Task Force on Practice Guidelines. Am Heart Assoc J.

[8] Mylotte D, Lefèvre T, Søndergaard L, Watanabe Y, Modine T, Dvir D, Bosmans J, Tchétché D, Kornowski R, Sinning J-M, Thériault-Lauzier P, O'Sullivan CJ, Barbanti M, Debry N, Buithieu J, Codner P, Dorfmeister M, Martucci G, Nickenig G, Wenaweser P, Tamburino C, Grube E, Webb JG, Windecker S, Lange R, Piazza N (2014). Transcatheter aortic valve replacement in bicuspid aortic valve disease. J Am Coll Cardiol.

[9] Hayashida K, Bouvier E, Lefèvre T, Chevalier B, Hovasse T, Romano M, Garot P, Watanabe Y, Farge A, Donzeau-Gouge P, Cormier B, Morice M-C (2013). Transcatheter aortic valve implantation for patients with severe bicuspid aortic valve stenosis. Circ Cardiovasc Interv.

[10] Kochman J, Huczek Z, Ścisło P, Dabrowski M, Chmielak Z, Szymański P, Witkowski A, Parma R, Ochala A, Chodór P, Wilczek K, Reczuch KW, Kubler P, Rymuza B, Kołtowski Ł, Ścibisz A, Wilimski R, Grube E, Opolski G (2014). Comparison of one and 12-month outcomes of transcatheter aortic valve replacement in patients with severely stenotic bicuspid versus tricuspid aortic valves (Results from a Multicentre Registry). Am J Cardiol.

[11] Bauer T, Linke A, Sievert H, Kahlert P, Hambrecht R, Nickenig G, Hauptmann KE, Sack S, Gerckens U, Schneider S, Zeymer U, Zahn R (2014). Comparison of the effectiveness of transcatheter aortic valve implantation in patients with stenotic bicuspid versus tricuspid aortic valves (from the German TAVI Registry). Am J Cardiol.

[12] Gooley R, Lockwood S, Antonis P, Meredith IT (2013). The SADRA Lotus Valve System: a fully repositionable, retrievable prosthesis. Miner Cardioangiol.

[13] Meredith IT, Hood KL, Haratani N, Allocco DJ, Dawkins KD (2012). Boston scientific lotus valve. EuroIntervention.

[14] Grygier M, Araszkiewicz A, Lesiak M, Oko-Sarnowska Z, Trojnarska O, Olasińska-Wiśniewska A, Misterski M, Buczkowski P, Jemielity M, Grajek S (2015). The new generation is coming. Percutaneous implantation of the fully repositionable Lotus® aortic valve prosthesis: the first Polish experience. Kardiol Pol.

[15] Meredith Am IT, Walters DL, Dumonteil N, Worthley SG, Tchétché D, Manoharan G, Blackman DJ, Rioufol G, Hildick-Smith D, Whitbourn RJ, Lefèvre T, Lange R, Müller R, Redwood S, Allocco DJ, Dawkins KD (2014). Transcatheter aortic valve replacement for severe symptomatic aortic stenosis using a repositionable valve system: 30-day primary endpoint results from the REPRISE II study. J Am Coll Cardiol.

[16] Sievers H-H, Schmidtke C (2007). A classification system for the bicuspid aortic valve from 304 surgical specimens. J Thorac Cardiovasc Surg.

[17] Grube E, Schuler G, Buellesfeld L, Gerckens U, Linke A, Wenaweser P, Sauren B, Mohr F-W, Walther T, Zickmann B, Iversen S, Felderhoff T, Cartier R, Bonan R (2007). Percutaneous aortic valve replacement for severe aortic stenosis in high-risk patients using the second- and current third-generation self-expanding CoreValve prosthesis: device success and 30-day clinical outcome. J Am Coll Cardiol.

[18] Webb JG, Chandavimol M, Thompson CR, Ricci DR, Carere RG, Munt BI, Buller CE, Pasupati S, Lichtenstein S (2006). Percutaneous aortic valve implantation retrograde from the femoral artery. Circulation.

[19] Kappetein AP, Head SJ, Généreux P, Piazza N, van Mieghem NM, Blackstone EH, Brott TG, Cohen DJ, Cutlip DE, van Es G-A, Hahn RT, Kirtane AJ, Krucoff MW, Kodali S, Mack MJ, Mehran R, Rodés-Cabau J, Vranckx P, Webb JG, Windecker S, Serruys PW, Leon MB (2012). Updated standardized endpoint definitions for transcatheter aortic valve implantation: the valve academic research consortium-2 consensus document. J Am Coll Cardiol.

[20] Yousef A, Simard T, Webb J, Rodés-Cabau J, Costopoulos C, Kochman J, Hernández-Garcia JM, Chiam PT, Welsh RC, Wijeysundera HC, García E, Ribeiro HB, Latib A, Huczek Z, Shanks M, Testa L, Farkouh ME, Dvir D, Velianou JL, Lam BK, Pourdjabbar A, Glover C, Hibbert B, Labinaz M (2015). Transcatheter aortic valve implantation in patients with bicuspid aortic valve: A patient level multi-center analysis. Int J Cardiol.

[21] Yoon SH, Lefèvre T, Ahn JM, Perlman GY, Dvir D, Latib A, Barbanti M, Deuschl F, De Backer O, Blanke P, Modine T, Pache G, Neumann FJ, Ruile P, Arai T, Ohno Y, Kaneko H, Tay E, Schofer N, Holy EW, Luk NHV, Yong G, Lu Q, Kong WKF, Hon J, Kao HL, Lee M, Yin WH, Park DW, Kang SJ, Lee SW, Kim YH, Lee CW, Park SW, Kim HS, Butter C, Khalique OK, Schaefer U, Nietlispach F, Kodali SK, Leon MB, Ye J, Chevalier B, Leipsic J, Delgado V, Bax JJ, Tamburino C, Colombo A, Søndergaard L, Webb JG, Park SJ (2016). Transcatheter Aortic Valve Replacement With Early- and New-Generation Devices in Bicuspid Aortic Valve Stenosis. J Am Coll Cardiol.

[22] Adams DH, Popma JJ, Reardon MJ, Yakubov SJ, Coselli JS, Deeb GM, Gleason TG, Buchbinder M, Hermiller J, Kleiman NS, Chetcuti S, Heiser J, Merhi W, Zorn G, Tadros P, Robinson N, Petrossian G, Hughes GC, Harrison JK, Conte J, Maini B, Mumtaz M, Chenoweth S, Oh JK (2014). Transcatheter aortic-valve replacement with a self-expanding prosthesis. N Engl J Med.

[23] Tanaka R, Yoshioka K, Niinuma H, Ohsawa S, Okabayashi H, Ehara S (2010). Diagnostic value of cardiac CT in the evaluation of bicuspid aortic stenosis: comparison with echocardiography and operative findings. AJR Am J Roentgenol.

[24] Leber AW, Eichinger W, Rieber J, Lieber M, Schleger S, Ebersberger U, Deichstetter M, Vogel J, Helmberger T, Antoni D, Riess G, Hoffmann E, Kasel AM (2013). MSCT guided sizing of the Edwards Sapien XT TAVI device: impact of different degrees of oversizing on clinical outcome. Int J Cardiol.

[25] Barbanti M, Yang T-H, Rodés-Cabau J, Tamburino C, Wood DA, Jilaihawi H, Blanke P, Makkar RR, Latib A, Colombo A, Tarantini G, Raju R, Binder RK, Nguyen G, Freeman M, Ribeiro HB, Kapadia S, Min J, Feuchtner G, Gurtvich R, Alqoofi F, Pelletier M, Ussia GP, Napodano M, de Brito FS, Kodali S, Nørgaard BL, Hansson NC, Pache G, Canovas SJ, Zhang H, Leon MB, Webb JG, Leipsic J (2013). Anatomical and procedural features associated with aortic root rupture during balloon-expandable transcatheter aortic valve replacement. Circulation.

[26] Gurvitch R, Tay EL, Wijesinghe N, Ye J, Nietlispach F, Wood DA, Lichtenstein S, Cheung A, Webb JG (2011). Transcatheter aortic valve implantation: lessons from the learning curve of the first 270 high-risk patients. Catheter Cardiovasc Interv.

[27] Schroeter T, Linke A, Haensig M, Merk DR, Borger MA, Mohr FW, Schuler G (2012). Predictors of permanent pacemaker implantation after Medtronic CoreValve bioprosthesis implantation. Europace.

[28] Yang T-H, Webb JG, Blanke P, Dvir D, Hansson NC, Nørgaard BL, Thompson CR, Thomas M, Wendler O, Vahanian A, Himbert D, Kodali SK, Hahn RT, Thourani VH, Schymik G, Precious B, Berger A, Wood DA, Pibarot P, Rodés-Cabau J, Jaber WA, Leon MB, Walther T, Leipsic J (2015). Incidence and severity of paravalvular aortic regurgitation with multidetector computed tomography nominal area oversizing or undersizing after transcatheter heart valve replacement with the sapien 3: a comparison with the sapien XT. JACC Cardiovasc Interv.

[29] Kochman J, Rymuza B, Huczek Z (2015). Transcatheter aortic valve replacement in bicuspid aortic valve disease. Curr Opin Cardiol.

[30] Schaefer A, Linder M, Treede H, Deuschl F, Schofer N, Seiffert M, Schneeberger Y, Blankenberg S, Reichenspurner H, Schaefer U, Conradi L (2016). Applicability of next generation balloon-expandable transcatheter heart valves in aortic annuli exceeding formally approved dimensions. Clin Res Cardiol.

[31] Gooley RP, Talman AH, Cameron JD, Lockwood SM, Meredith IT (2015). Comparison of self-expanding and mechanically expanded transcatheter aortic valve prostheses. JACC Cardiovasc Interv.

[32] Bagur R, Kwok CS, Nombela-Franco L, Ludman PF, de Belder MA, Sponga S, Gunning M, Nolan J, Diamantouros P, Teefy PJ, Kiaii B, Chu MWA, Mamas MA (2016). Transcatheter aortic valve implantation with or without preimplantation balloon aortic valvuloplasty: a systematic review and meta-analysis. J Am Heart Assoc.

[33] Blackman DJ, Van Gils L, Bleiziffer S, Gerckens U, Petronio AS, Abdel-Wahab M, Werner N, Khogali SS, Wenaweser P, Wöhrle J, Soliman O, Laborde JC, Allocco DJ, Meredith IT, Falk V, Van Mieghem NM (2019). Clinical outcomes of the lotus valve in patients with bicuspid aortic valve stenosis: an analysis from the RESPOND study. Catheter Cardiovasc Interv.

